# Mitochondrial NAD^+^-dependent malic enzyme from *Anopheles stephensi: *a possible novel target for malaria mosquito control

**DOI:** 10.1186/1475-2875-10-318

**Published:** 2011-10-26

**Authors:** Jennifer Pon, Eleonora Napoli, Shirley Luckhart, Cecilia Giulivi

**Affiliations:** 1 Department of Molecular Biosciences, School of Veterinary Medicine, University of California Davis, Davis, CA 95616, USA; 2 M.I.N.D. Institute, School of Medicine, University of California Davis, Sacramento, CA 95817, USA; 3 Department of Medical Microbiology and Immunology, School of Medicine, University of California Davis, Davis, CA 95616, USA

**Keywords:** malaria, mitochondria, bioenergetics, metabolism, inhibitors, mosquitoes

## Abstract

**Background:**

*Anopheles stephensi *mitochondrial malic enzyme (ME) emerged as having a relevant role in the provision of pyruvate for the Krebs' cycle because inhibition of this enzyme results in the complete abrogation of oxygen uptake by mitochondria. Therefore, the identification of ME in mitochondria from immortalized *A. stephensi *(ASE) cells and the investigation of the stereoselectivity of malate analogues are relevant in understanding the physiological role of ME in cells of this important malaria parasite vector and its potential as a possible novel target for insecticide development.

**Methods:**

To characterize the mitochondrial ME from immortalized ASE cells (Mos. 43; ASE), mass spectrometry analyses of trypsin fragments of ME, genomic sequence analysis and biochemical assays were performed to identify the enzyme and evaluate its activity in terms of cofactor dependency and inhibitor preference.

**Results:**

The encoding gene sequence and primary sequences of several peptides from mitochondrial ME were found to be highly homologous to the mitochondrial ME from *Anopheles gambiae *(98%) and 59% homologous to the mitochondrial NADP^+^-dependent ME isoform from *Homo sapiens*. Measurements of ME activity in mosquito mitochondria isolated from ASE cells showed that (*i*) *V_max _*with NAD^+ ^was 3-fold higher than that with NADP^+^, (*ii*) addition of Mg^2+ ^or Mn^2+ ^increased the *V_max _*by 9- to 21-fold, with Mn^2+ ^2.3-fold more effective than Mg^2+^, (*iii*) succinate and fumarate increased the activity by 2- and 5-fold, respectively, at sub-saturating concentrations of malate, (*iv*) among the analogs of L-malate tested as inhibitors of the NAD^+^-dependent ME catalyzed reaction, small (2- to 3-carbons) organic diacids carrying a 2-hydroxyl/keto group behaved as the most potent inhibitors of ME activity (e.g., oxaloacetate, tartronic acid and oxalate).

**Conclusions:**

The biochemical characterization of *Anopheles stephensi *ME is of critical relevance given its important role in bioenergetics, suggesting that it is a suitable target for insecticide development.

## Background

Recently, several pathways for energy production have been identified in mitochondria from *Anopheles stephensi *[1], a well-studied *Anopheles *species in the investigation of malaria transmission [2]. The mitochondria-dependent energy pathways mainly use proline, pyruvate, α-glycerophosphate, and acyl-carnitine derivatives as suitable substrates. Proline is also the main substrate for flight metabolism in the tsetse fly [3], the mosquito *Aedes aegypti *[4] as well as other insects [5]. About 20% of the glutamate produced by proline oxidation is in turn oxidized by glutamate dehydrogenase [6], whereas the remainder undergoes transamination by reaction with pyruvate and the resulting alanine accumulates as the proline is utilized. The 2-oxoglutarate formed by transamination is further metabolized by the Krebs' cycle. Originally pyruvate was thought to be produced from oxaloacetate by an oxaloacetate decarboxylase [7], but this enzyme was later localized in the cytoplasm whereas proline oxidation and subsequent reactions all take place in the mitochondria [6], consistent with previous studies [1]. Mitochondria of cultured cells [ASE cell line (*A. stephensi *Mos. 43 cell line)] from *A*. *stephensi*, as well as flight muscle mitochondria of a beetle (*Popillia japonica*), which also have the ability to oxidize proline at a high rate, have been shown to contain an unusually active malic enzyme [8]. The latter species utilizes NAD^+ ^preferentially as a coenzyme and presumably produces pyruvate by the oxidative decarboxylation of malate [8]. This mitochondrial enzyme in insects may have a critical role in the replenishment of pyruvate for either transamination or Krebs' cycle.

Malic enzyme (ME; EC 1.1.1.39) catalyses the reversible oxidative decarboxylation of *L*-malate to pyruvate and CO_2 _with the concomitant reduction of the cofactor NAD^+ ^or NADP^+ ^[9-11]. The enzyme requires divalent cations (Mg^2+^, Mn^2+^, or others) in the catalysis of this reaction. ME activity was first isolated from pigeon liver [12] and has since been found in most living organisms, from bacteria to humans. Most MEs are homotetramers, with monomers containing 550 amino acids and having molecular weights of 60 kDa. The amino acid sequences of MEs are highly conserved across all studied organisms, but they lack recognizable homology to other proteins, including other oxidative decarboxylases. The wide distribution of ME activity in nature and the high degree of sequence conservation are consistent with the important biological functions of these enzymes, such as photosynthesis in C4 plants and even some C3 plants [13] and biosynthesis of fatty acids and steroids in liver and adipose tissues in animals. In mammals, three isoforms of ME have been identified--cytosolic NADP^+^-dependent ME (ME-1; [14]), mitochondrial NADP^+^-dependent ME (ME-3; [15]), and mitochondrial NAD(P)^+^-dependent ME (ME-2; [10]), which can use either NAD^+ ^and NADP^+ ^as a cofactor (dual specificity), but prefers NAD^+ ^under physiological conditions. In invertebrates, and in particular in insects, unusually high activity of NAD^+^-linked malic enzyme has been reported in flight muscle mitochondria of the beetle *Popillia japonica *[8] and from the tsetse fly and other insects [16].

Based on previous reports [1], ASE mitochondrial ME emerged as having a relevant role in the provision of pyruvate for the Krebs' cycle because the chemical inhibition of this enzyme resulted in the complete abrogation of oxygen uptake by mitochondria. Therefore, the identification of ME in ASE mitochondria and the investigation of the stereoselectivity of malate analogues are relevant in understanding the physiological role of ME in cells of this important malaria parasite vector and its potential as a possible novel target for insecticide development.

## Methods

### Chemicals

Organic acids were purchased from Sigma Chemical Co. (St. Louis, USA). All reagents were of analytical grade.

### Cell maintenance

The immortalized *A. stephensi *ASE cell line was grown in modified Eagle's minimal essential medium ("E5") supplemented with glucose, L-glutamine, vitamin solution, nonessential amino acids, penicillin and streptomycin, and 5% heat-inactivated fetal bovine serum at 28°C with 5% CO_2 _as described [1]. The population doubling time of these cells is approximately 18-20 h. The cells were split 1:10 into E5 medium and grown in 50 ml culture flasks until confluent. These flasks were used to seed 500-ml culture flasks to prepare ~2 billion cells for mitochondria preparation. Cells harvested for mitochondria preparation were gently pipetted, resuspended in the medium, and transferred to 50-ml tubes. Cells were pelleted by centrifugation at 800 g for 5 min. The supernatant was removed, and the cells were resuspended in a small amount of medium by gentle pipetting and transferred to a sterile holding tube on ice. This cycle was repeated, with collection of the concentrated cells into one tube, until all flasks were processed.

### Isolation of mitochondria

Cells were centrifuged for 1 min at 500 g at 4°C and mitochondria were isolated from pelleted cells as described [1]. The pellet was weighed and MSHE buffer was added at a ratio of 3 ml of MSHE buffer (220 mM mannitol, 70 mM sucrose, 0.5 mM EGTA, 0.1% fatty acid-free bovine albumin, and 2 mM HEPES, pH 7.4) per 1 g of cells. The cells were disrupted by gentle homogenization, centrifuged at 600 g for 5 min at 4°C, the pellet was discarded, and the supernatant was centrifuged at 10,300 g for 10 min at 4°C. The pellet, which is rich in mitochondria, was resuspended in a small volume of MSHE. Using this procedure the yield was 7.5 ± 0.5 μg mitochondrial protein/10^6 ^cells. Protein concentration was determined by using the BCA Protein Assay (Pierce).

### Enzymatic assays

The ME enzymatic assay was performed using a method outlined by [17] with the following modifications. Mosquito mitochondria were homogenized in MSHE containing 2 mM mercaptoethanol. In a 1 ml cuvette, 2-μg/ml antimycin, 1 mM L-malate, 0.3 mM NAD^+^, 50 mM HEPES (pH 7.8), and 3 mM of MnCl_2 _(unless indicated otherwise) were added. The reaction was initiated with the addition of 40 μg of mosquito mitochondrial lysate protein. The change in absorbance was measured using the Cary 1E Spectrophotometer at 340 nm for 2-3 minutes (control). The ME specific activity was calculated for each trace utilizing the extinction coefficient for NADH at 340 nm (6.22 mM^-1 ^cm^-1^). Each L-malate analogue was added to the reaction mixture at the indicated concentrations and the change in absorbance was measured for another 3-5 minutes.

### Mass spectrometry analysis, protein identification, and confirmation of *A. stephensi *encoding sequence

LC-MS/MS analyses were performed at the Proteomics Facility of the University of California Davis Genome Center. Tandem mass spectra were extracted by BioWorks version 3.3. Charge state deconvolution and deisotoping were not performed. All MS/MS samples were analysed using X! Tandem [18,19]. X! Tandem was set up to search the Ensemble *A. gambiae *protein database (13,740 entries) assuming the digestion enzyme trypsin. X! Tandem was searched with a fragment ion mass tolerance of 0.40 Da and a parent ion tolerance of 1.8 Da. Iodoacetamide derivative of cysteine was specified in X! Tandem as a fixed modification. Deamidation of Asn and Gln, oxidation of Met and Trp, sulphone of Met, Trp oxidation to formylkynurenin of Trp and acetylation of the *N*-terminus were specified in X! Tandem as variable modifications. Scaffold (version Scaffold-3_00_08) was used to validate MS/MS based peptide and protein identifications. Peptide identifications were accepted if they could be established at greater than 90.0% probability as specified by the Peptide Prophet algorithm [20]. Protein identifications were accepted if they could be established at greater than 99.0% probability and contained at least 2 identified peptides. Protein probabilities were assigned by the Protein Prophet algorithm [21]. Proteins that contained similar peptides and could not be differentiated based on MS/MS analysis alone were grouped to satisfy the principles of parsimony.

To identify the full-length coding sequence for *A. stephensi *ME, the *A. gambiae *ME amino acid sequence (Q7QB64) was used as a query by Dr. Zhijian (Jake) Tu (Virginia Tech) to identify homologous sequence in the June 2010 unpublished draft of the *A. stephensi *assembly by TBLASTN (e-value cutoff 1e^-7^). The region with the best match plus 1-kb flanking sequences on either side were retrieved.

### Statistical analyses

The experiments were run in duplicate or triplicate and repeated two times in independent experiments. Data were expressed as mean ± SEM. The data were evaluated by using the *t*-test (StatSimple v2.0.5; Nidus Technologies, Toronto, Canada) with *p *≤ 0.05 considered as statistically significant.

## Results and discussion

### Identification of the mitochondrial ME from *A. stephensi*

**Figure 1:**
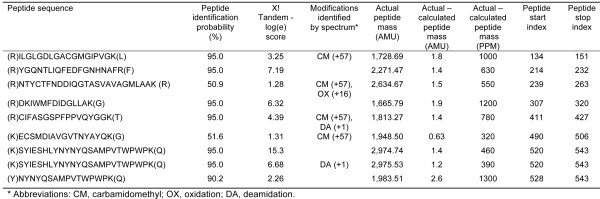
**Mass spectrometry analyses of ASE ME**. Mass spectrometry results from MudPIT analyses indicating the peptide sequences, identification probability to *A. gambiae *Q7QB64, peptide modifications identified by spectrum (carboxymethylated or CM, methionine sulfoxide or OX, and deaminated or DA), calculated and actual peptide masses, and peptide start and end amino acid from the above indicated *A. gambiae *sequence.

To characterize the ME in *A. stephensi *cells, Multidimensional Protein Identification Technology (MudPIT) was used. This technique involves digesting mosquito proteins with trypsin and separating the resulting peptides with two liquid column chromatography steps: the first being a strong cationic exchange, and the second being reversed-phase HPLC. As the peptides elute from the second column, they are sprayed into a linear ion trap mass spectrometer. The first MS scan assigned each peptide a mass/charge ratio. The most intense peptide signals are then fragmented in a second MS/MS scan, which assigns each peptide a unique "fingerprint" (Figure 1). The fingerprints are then analysed against bioinformatics databases (NCBI) that revealed the protein's identity.

**Figure 2:**
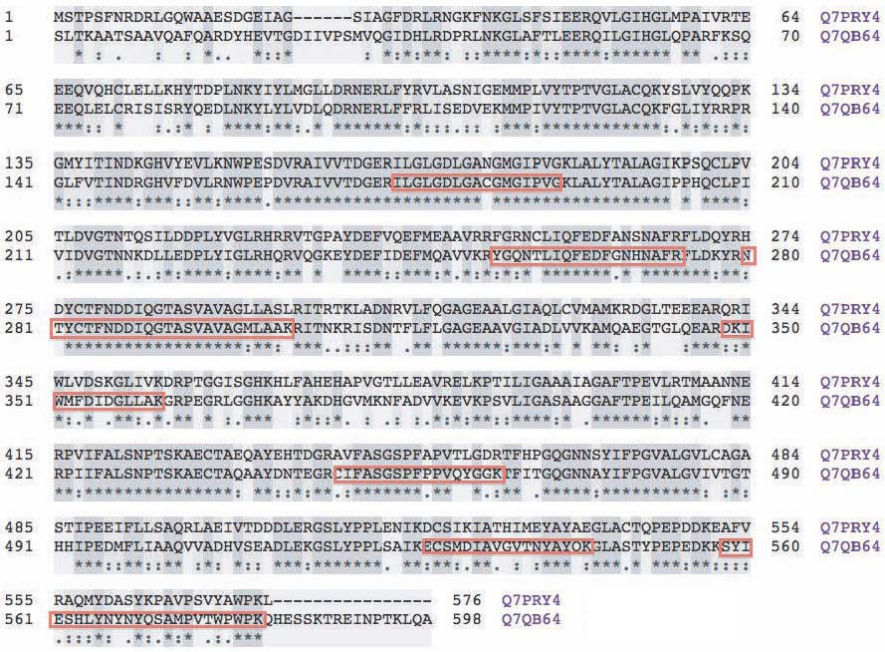
**Protein alignments of *A. gambiae *MEs**. Sequence alignment of the two ME proteins from *Anopheles gambiae *(Q7PRY4 and Q7QB64). Sequence alignments of the two ME proteins (sequences as reported in the SwissProt database; last visited on April 13, 2011). The alignments were generated using CLUSTAL [55]. Boxed amino acids indicate the peptides identified by mass spectrometry from Figure 1.

Six out of seven unique peptides had a ≥ 90% match with one of two MEs reported for *A. gambiae *(Q7QB64; Figure 1); only one of these peptides showed a peptide identification probability of approximately 51%. These peptides provided 26% protein coverage (148 amino acids out of 572; Figure 2). Given the high homology of these peptides to *A. gambiae *Q7QB64 (Figure 2), this sequence was used to query the *A. stephensi *genome assembly. This analysis retrieved the homologous *A. stephensi *ME sequence (Additional file 1). The predicted *A. stephensi *protein sequence (Additional file 1) revealed a 98% homology (99% positives) with *A. gambiae *ME (Q7QB64; Additional file 1).

BLAST query of the complete amino acid sequence of *A. stephensi *ME to the non-redundant NCBI database revealed high identity to MEs from mosquitoes (*A. gambiae 96%, Anopheles darlingi *94%, *Aedes aegypti *88%, *Culex pungens *87%), flies (*Drosophila *spp. 75%, *Glossina *spp. 62%), ants (*Harpegnathos *spp. 74%; *Camponotus *spp. 74%), lice (*Pediculus *spp. 73%), moth (*Bombyx *spp. 64%), and a nematode (*Ascaris suum *54%). BLAST query against the human database revealed a 59% identity to the human mitochondrial NADP^+^-dependent ME (or ME-3), followed by a 57% and a 55% identity to the human cytosolic ME-1 and the human mitochondrial ME-2, respectively. The lower homology of the *A. stepephensi *ME with the human mitochondrial counterpart was not due to the mitochondrial targeting sequence because its removal from the human isoform and realignment of the mature protein with the mosquito protein did not change the identity (59%), similarity (75%) or score (1742). Of note, the two human mitochondrial ME isoforms share only 54% of their amino acids, which is the same range of homology when comparing human MEs with *A. stephensi *ME (this study) or from maize chloroplasts (47%). By comparison the highly homologous *A. gambiae *ME (Q7QB64) is 59% identical to the human ME-3, 57% to human ME-1, and 55% to human ME-2, whereas the other ME from *A. gambiae *(Q7PRY4) has 60% identity to the human ME-1, 58% to ME-3, and 53% to ME-2. As in *A. gambiae*, two MEs have been identified for *A. darlingi*, in which one of them (E3WUS4) is 62% identical to the human ME-1, while the other ME (E3WW73) is 60% identical to the human ME-3. Thus, for these mosquito species, no ME-2 ortholog (mitochondrial and NAD^+^-dependent) is apparent. In agreement with these findings, only one mitochondrial ME from *A. stephensi *was detected in the performed MudPIT analyses and genome sequence analyses. However, a possible *A. stephensi *ME paralog with approximately 60% identity to *A. gambiae *ME (Q7QB64) was detected (Dr. Zhijian Tu, personal communication). While this encoded protein is potentially interesting, we have focused on the highly homologous *A. stephensi *ME here.

**Figure 3:**
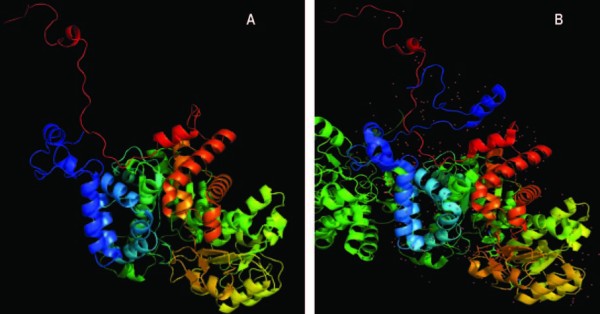
**Three-dimensional structures of *A. stephensi *and *Ascaris suum *MEs**. The 3D structure of *A. stephensi *ME (A) was obtained through modelling from the primary sequence from Additional file 1 and the use of the modeling software I-TASSER (results shown in Additional file 2). The 3D structure of *Ascaris suum *ME was obtained from the protein database and corresponds to the accession number 1o0sA. Both pdb files were visualized using PyMol 1.4.1 [56].

With the primary sequence of the *A. stephensi *ME, a high-quality prediction of the 3D structure and biological function of the ME was performed by using a template-based modelling platform [22,23]. The results of this modelling (Additional file 2) revealed that the 3D structure of *A. stephensi *ME (with a C-score of 1.985) was closely related to that of *Ascaris suum *(1o0sA; TM score 0.9645; RMSD 1.43, identity 0.53; coverage 99%; Figure 3). This 3D model of *A. stephensi *ME was based on templates from human ME-1 (2aw5B), human ME-2 (1pj3A and 1gz4A) and *Ascaris suum *(1llqA and 1o0sA; Additional file 2). The modelling results also predicted the binding site for various ligands (Additional file 2). In particular, the predicted binding sites for Mn^2+^, oxalate and NADP^+ ^resemble those in pig liver ME-1, whereas those for tartronate, malate, fumarate and Mg resembled those in human ME-2. The binding site for tartronate was also similar to that defined for *Ascaris suum *ME.

To gain insight in the activity of ME, enzymatic parameters were evaluated in mosquito mitochondria isolated from ASE cells. The production of NAD(P)H was followed in the presence of malate and NAD(P)^+^. The *K*_m _value for L-malate was calculated as a function of total concentrations of the substrate and it was found to be 0.12 mM at the pH of 7.8 (Table 1). A low activity was obtained in the absence of cofactors (Mg^2+ ^or Mn^2+^), but the addition of either metal increased significantly the maximum activity (9 to 21-fold); however, Mn^2+ ^was a better cofactor than Mg^2+ ^(2.3-fold of Mg^2+ ^alone; Table 1). The specific activity of the ASE ME enzyme was significantly higher than that of the other two human mitochondrial isoforms: the specific activities of the human mitochondrial NADP^+^-ME and NAD^+^-ME have been reported as 12 U/mg protein (1 U or unit = 1 μmol/min) and 35 U/mg protein, respectively, whereas the activity of the human cytosolic NADP^+^-ME is 40 to 55 U/mg [15]. ASE ME could utilize either NAD^+ ^or NADP^+^; however, *V_max _*with NAD^+ ^was 3-times higher than that with NADP^+ ^(Table 1), consistent with the predictions made from the protein similarity search (*vide supra*) and structure (*vide infra*).

**Table 1:** Effect of cofactors on V_max _of ME activity

Addition	*K_m _*for malate (mM)	*V_max_* nmol × (min × mg protein)^-1^	*p*-value to controls
None	-	3 ± 1	-

MgCl_2 _(3 mM)	-	24 ± 2	0.02

MnCl_2 _(3 mM)	0.12	56 ± 3 (20 2)	0.01

All kinetic parameters were obtained with NAD^+ ^except the *V_max _*obtained with NADP^+ ^(value in parentheses). The *V_max _*values were expressed as mean ± SEM. All other experimental details are described in the Methods.

**Figure 4:**
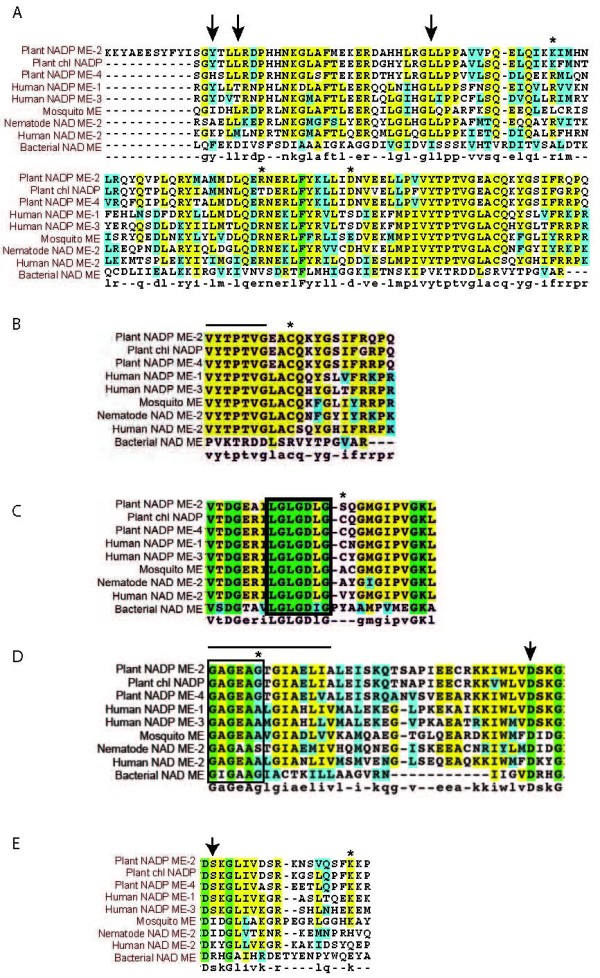
**Analyses of protein sequence alignments of various MEs**. Sequence alignments of maize (NADP^+^-ME-2, chloroplastic NADP^+^-ME, and NADP^+^-ME-4), human (ME-1, ME-2, and ME-3), nematode (NAD^+^-ME-2), bacterial (NAD^+^-ME) and *A. stephensi *ME (mosquito ME). The amino acid sequences of the ME isoforms were analyzed by BLAST against the SwissProt database, and the alignments were generated using CLUSTAL [55]. The amino acid residues highlighted in grey share high homology whereas those in bold are identical. Key amino acids discussed in the text are shown with asterisks, arrows or bars (see text for full description). (A) Sites involved in fumarate activation; (B) Malate binding site; (C) ADP binding site; (D) NAD(P)^+ ^binding domain and sites associated with NAD^+ ^versus NADP^+ ^preference; (E) Other sites also associated with NAD^+ ^versus NADP^+ ^preference. For complete protein nomenclature see Additional file 3.

**Figure 5:**
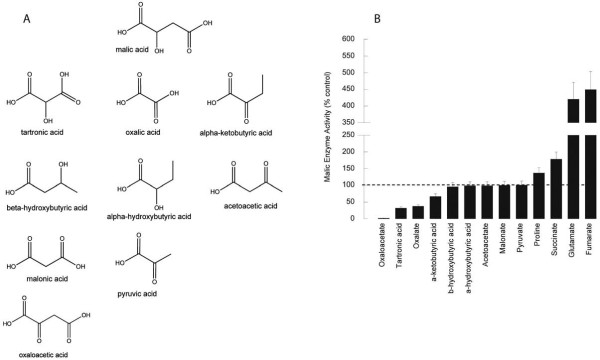
**Effect of L-malate analogues on ME activity**. A. Chemical structure of L-malate and structural analogues. B. Effect of L-malate analogues on ASE mitochondrial ME enzymatic activity. The activity of ME was evaluated in the absence and presence of the following inhibitors/compounds at saturating concentrations of all other cofactors and substrates except for succinate and fumarate which are shown at subsaturating concentrations of malate (0.5 mM): 1 mM oxaloacetate; 5 mM tartronic acid; 1 mM oxalate; 2.5 mM alpha-ketobutyrate; 2.5 mM beta-hydroxybutyrate; 2.5 mM alpha-hydroxybutyrate; 2.5 mM acetoacetate; 2.5 mM malonate; 10 mM pyruvate; 10 mM proline; 10 mM succinate; 10 mM glutamate; and 5 mM fumarate.

It has been shown that the presence of basic residues at positions 67 and 91 of human ME-2 are apparently critical for fumarate-dependent activation [24] (Figure 4A, residues marked with asterisks). All three human isoforms contain Arg at the equivalent positions [24] but only ME-2 is activated by fumarate. Thus, other factors may contribute to this activation [24]. The mutation of the amino acid residue Asp-102 had a significant effect on the fumarate-mediated activation of human ME-2 [25]. At this position, the human ME-1 and ME-3 isoforms have a Ser residue and they do not show any increase in activity with fumarate. The mosquito ME has a Glu at this position, and the *Ascaris suum *ME has an Asp, suggesting that these isoforms, like the human ME-2, may be activated by fumarate by conserving the negative charge at this position. On the other hand, in the mosquito ME, Leu occupies the equivalent position of Arg-67 (all human isoforms and the nematode ME have an Arg in this position), suggesting that mosquito ME should not be activated by fumarate. In addition to Arg-67, it has been reported that in the nematode mitochondrial ME, the residues that are involved in the binding of fumarate are Ala-78, Leu-81 and Leu-105 (Figure 4A, rows indicated with arrows), which in *A. stephensi *ME are occupied by Ile, Leu and Leu in agreement with the nematode isoform, which is activated by fumarate. In agreement with this last prediction, succinate and fumarate increased the activity by 2- to 5-fold, respectively, at sub-saturating concentrations of malate (Figure 5B), showing no effect at saturating ones (not shown). Other amino acids, such as Pro, had a moderate effect on enzyme activity up to concentrations of 10 mM (1.4-fold). Surprisingly, Glu was found to be a potent activator increasing ASE ME activity by 4-fold at saturating concentrations of malate (Figure 5B).

One very highly conserved region defined as a part of a malate-binding domain is shown in Figure 4B. This region has been identified on the basis of inhibition experiments carried out on NADP^+^-dependent ME with the competitive inhibitor bromopyruvate [15]. Indeed, a highly conserved Cys (Figure 4B, asterisk) and the preceding sequence VYTPTVG (Figure 4B, bar) are present in this domain in human, nematode, plant and *A. gambiae *and *A. stephensi *mosquito isoforms (Figure 4B), suggesting that the same type of binding occurs in the mosquito isoforms. However, recently the role of this Cys has been challenged based on crystallography data [24]. This study indicated that this residue is about 13 Å away from the substrate analog oxalate suggesting that the inhibition of substrate binding upon chemical modification of this Cys is an indirect effect.

The proposed ADP binding beta-alpha-beta fold is shown which contains a characteristic arrangement of Gly residues and nonpolar and hydrophilic amino acids at specific positions (Figure 4C, boxed amino acids; [26]). A highly conserved Cys residue (or the highly similar Ser in plant NADP ME-2; Figure 4C, asterisk) observed in this region is present in all NADP^+^-dependent malic enzymes [27] but is absent from the NAD^+^-dependent malic enzymes (human ME-2, nematode and mosquito isoforms). Therefore the presence or absence of this Cys or Ser residue in this binding fold apparently distinguishes the preference for NADP^+ ^over NAD^+ ^as suggested before by others [28].

The fingerprint region of NAD(P)^+^-binding sites is characterized by a Gly-rich sequence (Gly-X-Gly-X-X-Gly; Figure 4D, boxed amino acids), which is the phosphate binding consensus [29,30]. The first Gly allows the tight turn of the main chain, the second allows a close contact to the diphosphate of NAD(P)^+^, and the third is important for the close packing of the secondary structure [29,31]. This third Gly is replaced by larger amino acids (Ala or Ser) in enzymes that utilize NADP^+ ^(e.g., glutathione reductase, thioredoxin reductase) because it is believed that disrupting the close packing of the secondary structure allows the structure to accommodate the phosphate moiety of NADP^+ ^[29]. Furthermore, protein engineering of glutathione reductase, in which Ala-179 was replaced by a Gly residue at an equivalent position in this enzyme, both in single as well as in multiple substitutions, caused NAD^+ ^binding preference over that of NADP^+ ^[32]. However, this reasoning does not seem to apply to all MEs listed in Figure 4D (position marked with asterisk). In particular, all human MEs have a third Gly regardless of the cofactor preference, indicating that other factors are contributing to the cofactor preference. In support of this statement, Gly residues in the boxed segments shown in Figures 4C &4D when replaced by Val residues rendered abortive mutants of maize C_4 _NADP^+^-ME [31]. This is consistent with the three-dimensional model obtained for the maize C_4 _NADP^+^-ME showing that residues at both sites are part of the NADP^+^-binding site justifying the high degree of conservation among all NAD(P)^+^-ME [31].

It has been suggested that two Ala residues spaced by three amino acids (Gly-X-Gly-X-X-Ala-X-X-X-Ala; Figure 4D indicated with a bar) are more characteristic for NADP^+^-binding domains relative to NAD^+^-binding domains, whereas Gly residues were more consistently identified in NAD^+^-dependent enzymes [31,32]. However, all human ME isoforms, nematode mitochondrial ME and *A. stephensi *ME have an Ala-X-X-X-Ala segment, regardless of whether they use NADP^+ ^or NAD^+^.

Other residues may also affect binding specificity of NAD(P)^+ ^in MEs. It has been noted that all NADP^+^-dependent ME isoforms have a conserved Lys at Gln-362 in human ME-2 (Figure 4E, asterisk; [33]), whereas dual specificity MEs have heterogeneous residues at this position [34] (Figure 4E, asterisk). This observation, combined with mutational and modelling studies using pigeon ME-1, led to the supposition that in NADP^+^-dependent MEs, NADP^+ ^specificity is conferred by Lys at this position. Mitochondrial NAD^+^-MEs of *Ascaris *and other species may have no need to exhibit strict specificity for NAD^+ ^due to the high concentration of NAD^+ ^relative to NADP^+ ^within mitochondria, and thus the residue at the corresponding position would not necessarily be constrained to Lys. Indeed, in mosquito ME (this study), nematode ME, human ME-2 and bacteria ME, this sequence position corresponds to a Lys, His, Gln and Glu, respectively (Figure 4E, asterisk). Recent data clearly indicate that the Gln-362-Lys mutant of human ME-2 is a non-allosteric, non-cooperative and NADP^+^-specific enzyme, as is ME-1 [33]. Additionally, Gln-362-Lys is more sensitive to ATP, and the inhibition constant is smaller than that of wild-type [33]. Sequence alignments of the nucleotide-binding region among MEs have revealed that, in addition to Gln-362, Lys-346 is conserved among the NAD^+^-dependent malic enzymes (human ME-2; Figure 4E arrow), but in NADP^+^-dependent MEs (maize, human), this Lys is replaced by Ser (Figure 4E arrow). In the case of *A. stephensi *ME, this residue is replaced by Ile, as it is in the case of the nematode ME. Thus, in those isoforms with NADP^+^-only specificity, a Lys in position 362 (Figure 4E asterisk) and a Ser in 346 (Figure 4E arrow) are apparently necessary.

ATP acts as an active-site inhibitor of mitochondrial ME-2 following a competitive mechanism for NAD^+ ^and malate with *K_i _*values from 80 to 430 μM [24,35]. According to Hsieh *et al *[36], the presence of Lys-346 in mammalian ME-2 is critical for ATP inhibition (Figure 4E, arrow) because site-directed mutagenesis of this residue to Ala or Ser diminishes ATP-mediated inhibition. This result suggested that the positive charge is critical for ATP binding. As indicated above, in the human ME-1 and ME-3 as well as the plant MEs, in isoforms that are not inhibited by ATP, the equivalent positions for Lys-346 are occupied by Ser. In the mitochondrial nematode and mosquito MEs, Ile residues are present in these positions, thus no significant effect of ATP would be expected as it has been shown for the *Ascaris *ME [37,38]. In agreement with this prediction, kinetic results showed that ATP was not an inhibitor with respect to NAD^+ ^or L-malate in *A. stephensi *mitochondrial ME at the concentrations tested (20 to 1,000 μM; data not shown). The Arg-197, Arg-542 and Arg-556 residues in human ME-2 appear to be involved in the binding of ATP at the exosite [24]. These residues differ from those in human ME-3 or mosquito ME (human ME-3 = Gln, Tyr and Leu and mosquito ME = Gln, Thr and His), suggesting that this binding is not operational in these isoforms.

### Inhibitors of the *A. stephensi *mitochondrial ME

Several analogues of L-malate were tested as inhibitors of the NAD^+^-dependent ME catalyzed reaction (Figure 5A). Oxalate, oxaloacetate, tartronate and alpha-ketobutyrate were found to be inhibitors of the ME-catalyzed reaction, through a competitive mechanism with respect to the substrate (malate) concentration. Their *K_i _*values ranged from 0.1 (oxaloacetate and oxalate) to 0.6 μM (tartronate; Table 2). Alpha-hydroxybutyrate and the ketone bodies beta-hydroxybutyrate and acetoacetate did not exhibit any effect on the ME activity. The same outcome was obtained with either malonate or pyruvate (Figure 5B).

**Table 2:** Inhibition constants of various malate analogues

Compound	*K_i _*(μM)
Oxaloacetate	0.12

Oxalate	0.15

Tartronate	0.61

Alpha-ketobutyrate	n.d.

Inhibition studies were performed by varying the concentrations of L-malate from 0.1 mM (subsaturating) to up to 1 mM (saturating) while the analogue (chosen based on its inhibitory activity from Figure 3B) was held at several fixed concentrations around its inhibition constant. Concentrations of the other components in the assay mixture were held at saturation. Reciprocal velocities were plotted as a function of reciprocal substrate concentrations and all plots were linear in the range of substrate concentrations assayed (0.1-0.5 mM). The lines were calculated from the fits of the experimentally determined values to the appropriate kinetic parameters. Abbreviations: n.d, not determined.

The catalysis by ME generally proceeds in two steps, namely the dehydrogenation of malate to produce oxaloacetate, and then the decarboxylation to produce pyruvate. In a proposed enzymatic catalysis based on structural data the crystallography data, the latter step is believed to occur through the formation of a keto-enol intermediate structurally similar to oxalate [39]. The strongest ME inhibitors for which *K*_i _were evaluated (i.e., oxalate, oxaloacetate, and tartronate) are consistent with this mechanism, for these compounds are either intermediates (i.e., oxaloacetate, oxalate) or structurally similar intermediates (tartronate or ketomalonate) that fit the tight active site of the enzyme [39]. Besides the role of size, these inhibitors are diacids. Thus, the presence of an extra carboxyl group appears to be necessary for binding the substrate (or inhibitor) to the enzyme, probably through an induced dipolar type bond with the side chains of Gln and Asn, as suggested by others [40]. In support of this rationale, the presence of the extra carboxylate in malate and oxaloacetate compared to alpha-hydroxybutyrate and alpha-ketobutyrate, respectively, yielded stronger inhibition of ME (Figure 5).

Within the diacids, the presence of a keto group in position 2 seemed to be an important factor in controlling the inhibitory effect of the compounds studied, for lack of this moiety in, for example, succinate or fumarate, resulted in a loss of the inhibitory effect. The change of the 2-keto group of the alpha-ketobutyric acid to 2-hydroxy (alpha-hydroxybutyrate) or the transfer to the 3-hydroxy (beta-hydroxybutyrate) resulted in negligible effect on the ME activity (Figure 3B). Thus, the keto group in position 2 seems essential for effective inhibition of ME, possibly by favoring π-π interactions with the nicotinamide ring of NAD^+ ^and/or hydrogen bonding with the side chain amide of Asn and the 2'-hydroxyl of the nicotinamide ribose [39]. These results suggest that the extent of inhibition was dependent on the size of the analogues (2 to 4-carbons), the presence of two carboxyl groups along with a 2-hydroxyl or 2-keto moiety important for binding of the substrate analogue to the enzyme. In a study performed with the enzyme from *Flaveria trinervia*, the presence of a group with a low *p*K value (6 to 6.6), probably an H residue, was responsible for the binding of the 2-hydroxyl of malate and transfer of the hydride to form the intermediate oxaloacetate [41]. These results suggest that the extent of inhibition was dependent on the size of the analogues (2 to 3-carbons), the presence of two carboxyl groups along with a 2-hydroxyl or 2-keto moiety important for binding of the substrate analogue to the enzyme.

The ME-3 gene is conserved in human, chimpanzee, dog, cow, mouse, rat, a variety of flies and mosquito species, *Caenorhabditis elegans, Arabidopsis thaliana*, and rice. The mammalian ME-3 isoform has a strong tissue-specific expression, mostly in organs with a low division rate (e.g., heart, skeletal muscle and brain; [15]) or with an involvement in steroid hormone biosynthesis (ovary and testes). High activity of ME-3 has also been reported in muscle of crustaceans and fish [42]. It has been previously reported that the immortalized cells from *A. stephensi*, from which the ME activity in this study was evaluated, resembled skeletal muscle cells [1]. Thus, the finding of a high activity of this mitochondrial enzyme is consistent with this previous study. Given that the reverse reaction (i.e., the carboxylation of pyruvate) was 12-times slower than the forward reaction (not shown), the role for ME-3 in the anaplerotic reaction (pyruvate to malate) of the Krebs' cycle is expected to be negligible.

A previous report indicated that addition of tartronic acid completely inhibited the oxygen consumption of Pro-supplemented phosphorylating mitochondria [1]. This suggests a critical role for ME in a pathway to provide NADH to the electron transport chain. The ME will then be activated with glutamate, succinate or fumarate as in the case when Pro or other suitable substrate becomes available. The physiological function of the inhibition by oxalate is not clear given the limiting amount of this substrate in mitochondria. If NADH levels are high and citrate concentration rises when switching from high to low work, this would result in an inhibition of citrate synthase and increases in oxalocetate, which in turn would inhibit ME, and reduce the supply of pyruvate and acetylCoA. An interesting feature of this isoform is that the optimum pH was 7.8, which is probably close to the physiological intramitochondrial pH value under phosphorylating conditions [43].

It is likely that a secondary role of this enzyme in mosquito mitochondria, based on the preferential use of NAD^+ ^over NADP^+^, is to provide NADPH for the detoxification of reactive oxygen radicals generated in mitochondria during respiration. Glutathione reductase activity requires NADPH to recycle oxidized glutathione for the glutathione peroxidase system [44], and mitochondrial NAD^+^-ME might contribute to the generation of reducing equivalents.

## Conclusions

The critical role of ME in the bioenergetics of mosquitoes along with the deeper understanding of its biochemistry and chemical requirements for inhibition, suggests that this enzyme could became a suitable target for insecticide development. In this regard, structural analogues of L-malate (especially C2 or C3 organic diacids with a 2-hydroxyl or 2-keto group) behaved as the most potent inhibitors of ME activity (e.g., oxaloacetate, tartronic acid and oxalate).

Enzymes significantly associated with insecticide resistance include esterases and cytochrome P450s involved in the oxidative catabolism of insecticides [45-47]. Mitochondrial Complex I inhibitors seem to circumvent the aforementioned resistance [5,48,49]. However, recent studies of pyrethroid-resistant *A. gambiae *demonstrated that exposure to permethrin induced strong upregulation of genes that encode subunits of Complex I [50] whereas the presence of MAP kinase Pmk1 and PKA conferred resistance to *Schizosaccharomyces pombe *to rotenone, a potent Complex I inhibitor [51]. In addition, whereas most cases of insecticide resistance were usually associated with mutations in the nuclear genome, a role for maternally-inherited mtDNA has emerged as a possible mediator of resistance to Complex I inhibitors and insecticides [47,52]. These observations suggest that Complex I-associated resistance may already occur in natural populations. Thus, this possibility should be considered when resistance to current insecticides and development of novel insecticides are evaluated. While direct studies of insecticide resistance are beyond the scope of the present study, it is important to bear in mind that inhibition of multiple mitochondrial targets (e.g., Complex I and ME) might result in synergistic effects or in attenuation of the development of cross-resistance as already observed when multiple, unrelated targets for insecticides are tested [53,54].

## List of abbreviations

ASE: *Anopheles stephensi *Mos. 43 cell line; ME: malic enzyme; ME-1: cytosolic NADP^+^-dependent ME; ME-2: mitochondrial NAD(P)^+^-dependent ME; ME-3: mitochondrial NADP^+^-dependent ME; MS: mass spectrometry; MS/MS: tandem MS; mtDNA: mitochondrial DNA; MudPIT: Multidimensional Protein Identification Technology

## Competing interests

Drs. Giulivi and Luckhart filed a disclosure and record of invention with the University of California Davis on 04/11/2008 entitled "Inhibitors of mosquito malic enzyme as insecticides" (UC Case #2008-673-1).

## Authors' contributions

JP carried out all enzymatic analyses and performed statistical analyses. EN supervised directly the technical work, performed statistical analyses and helped to draft the manuscript. SL provided all biological materials, contributed with substantial intellectual input in regards to the malaria field, and revised the manuscript critically. CG conceived of the study, participated in its design and coordination, performed all sequence alignment and analyses, protein modelling and interpretation, and wrote the manuscript. All authors read and approved the final manuscript.

## Supplementary Material

Additional file 1 **DNA and protein sequences for *A. stephensi *ME**. A. Nucleotide sequence for *A. stephensi *ME. B. Amino acid sequence deduced from DNA equence. C. Protein sequence producing significant alignment to *A. stephensi *ME performed with BLAST. Click here for file

Additional file 2 **Modeling results obtained with *A. stephensi *ME**. This file contains the results obtained with the simulation suite named I-TASSER performed with the primary amino acid sequence of *A. stephensi *ME. Click here for file

Additional file 3 **Additional information on the nomenclature of proteins listed under Figure 4**. This file contains all additional information (full name, short name, E.C. number, and species) in regards to the nomenclature of ME shown under Figure 4. Click here for file
