# Supplementary material for: Mitochondrial NAD+-dependent malic enzyme from Anopheles stephensi: a possible novel target for malaria mosquito control
Source: Malar J. 2011 Oct 26;10:318. doi: 10.1186/1475-2875-10-318 (PMC3228860; doi:10.1186/1475-2875-10-318)
Supplement: Additional file 1 — DNA and protein sequences for A. stephensi ME. A. Nucleotide sequence for A. stephensi ME. B. Amino acid sequence deduced from DNA equence. C. Protein sequence producing significant alignment to A. stephensi ME performed with BLAST. [file 1475-2875-10-318-S1.PDF]

Additional file 1

A. Gene sequence for malic enzyme from *Anopheles stephensi*

ACATGCGTACGAGAGGTGAAAAATCAACCACCCCACGTTCGATATTCGGTAGTAAACAAACCAACCAgcACCGtGCTCTGGGCActAATAACACGGCCTCGAT
CGATAATTtACACAGCGAAAAAAAAaaaGAAaGGTCACTTACCCACCCGGACGTCgCGTCAAGTGGGgTGATAAGGCGATTGTGCGAATCGTCGATAAATGAT
GTTagCACTTcGCaGAGACGATCCATCCACACGACCGTTTCGCTATAAAGTATGACCGCGTTGTCGCCTGACCGGATTTAGCGAGATTCGGACGCCCGTTTCC
AATCGCTGGATTATGCAAACAATTTAGTTGAAATGGCAATATCGCGTTACCGCACACATTACGGgCACCTCCATCGCTGTTTTACCCATCGTCCcTCGCCAAA
GGAATCTATTTTCAGGGACCTACTTCGATTAGCCGCGAGTGTTTGtACTTTTGTCAACCACCGCAACACAACCGTGGTTGACTATAAATAGAGCAGCAAGCGG
GAACGGCTCGAAACGGGACGTGATGTCTCCGAGTTCGCGCTtCGAAAACCTCGACCTTAGacaaaTGTTCGaGgCAGATCAGATCGCTGACAAtACAAAGACG
ATGGGCGGAAAATTGttCCCCCcGTGTATCCGTGTCCGCAAGGTTTtCTGCTTCGGGTGTGGGTCAGGCCCTTTGCTCGCTTCAAGGTGTTGCCCTGTTaGAA
CACATCCACGGCAAGGGCACAACAAAAGGGGTTGCTTTATTTTTATACGACGTTCTTGTCGGATTATTTAATGTCCAAaGGGTTCACCATCTCTCTCTCTCTC
gtCTTTCTCTTAttCGTTTTAGCCTGAACGCCAAACAGACCGTCGATGCGTTGCGGACGCTGGTCGTGACCGCATCGCCGAAGAAATCGATCACCAAAGCGGC
AACGTCGGCCGCCGTGCAAGCGATCCAGACGCGCGATTACCATGAGGTGACGGgCGACATCATCGTACCGTCGATGGTGCAGGGCATTGACCATTTGCGCGAT
CCGCGCCTCAACAAGGGTCTCGCATTTACGCTGGAAGAGCGTCAAATTCTGGGCATCCATGGGCTGCAGCCGGCCCGGTTTAAGTCGCAGGAGGAACAGCTGG
AaCTGTGCCGGATTTCGATCTCGCGCTACCAGGAAGATCTTAACAAGTACCTGTACCTGGTGGATCTGCAGgACCGTAACGAGCGGTTATTCTTCCGCCTGaT
CTCGGAGGACGTGGAGAAGATGATGCCGATCGTGTACACGCCCACGGTTGGGTTGGCGTGTCAAAAGTTCGGTTTGATCTATCGCCGACCGCGTGGtCTGTTC
GTGACGATTAACGATCGGGGCCATGTGTTCGATGTGCTGCGCAACTGGCCcgAACCGGACGTGCGTGCGATTGTCGTTACGGACGGGGAGCGCATtctGGGTT
TGGGTGATTTGGGTGCTTGCGGTATGGGCATTCCGGTCGGTAAGTTGGCGCTGTATACGGCGCTGGCCGGTATTCCACCGCACCAGTGTTTGCCGATCGTGAT
CGATGTGGGCACGAACAACAAGGATCTGCTGGAGGATCCGCTGTACATTGGGCTGCGGCATAAGCGTGTGCAGGGCAAGGAGTACGATGATTTCATCGACGAG
TtcATGCAGGCGGTCGTGAAGCGGTACGGGCAGAACACGCTCATCCAGTTCGAGGACTTTGGCAACCaCAATGCGTTCCGCTTTCTGGACAAGTACCGCAACA
CGTACTGCACGTTCAATGACGACATTCAGGGTACGgCTTCGGTGGCGGTGGCTGGACTGTTGGCCTCGAAGCGCATTACCAACAAGCGCATCTCGGAGAACAC
GTTCCTGTTtctCGGGGCGGGTGAAGCTGCCGTCGGTATTGCGGATCTGGTGGTGAAGGCGATGCAGGCGGAAGGCGTCGGCTTGCAGGAGGCTCGCGACAAG
ATTTGGATGTTCGACATTGATGGGCTGCTGGCGAAGGGCCGTCCGGAGGGTCGGCTCGGTGGACACAAAGCTTACTATGCCAAAGACCACGCTGTGATGAAGA
ACTTTGCCGACGTGGTGAAGGAGGTGAAGCCGTCGGTGTTGATCGGTGCGTCGGCTGCGGGTGGTGCGTTTACGCCGGAAATTCTGCAAGCCATGGGCCAGTT
CAATGAGCGTCCGATCATTTTCGCACTGTCGAATCCCACCTCGAAGGCGGAGTGCACGGCACAGGCGGCGTATGATAATACTGAGGTacGTGtgTACACGATA
TTGAtcgaGCCACAGCCGGaTTGATTGCGAATTGTTCTTTCCATTGCAGGGTCGCTGTATTTTTGCCTCTGGATCTCCGTTCGCCCCGGTAAAGTATGGCGGA
AAGACATACATTACGGGCCAGGGCAACAATGCGTACATCTTCCCGGGTGTGGCATTGGGTGTCATCGTTACCGGCACGCATCACATCCCCGAGGACATGTTCC
TcATTGCTgcccAAGTCGTCGCCGATCACGTGTGCGATGCGGATCTGGAGAAGGGCTCGCTCtACCCACCGCTCAGTGCCATTAAGGAATGCTCGATGGaTAT
TGCGGTCGGCGTTACGAACTACGCATATCAGAAGGGTAAGAGAGCGGGCGATTAGTAACGTCTtGCATGATGGATGTTACTCACCTTCCGTGTTTCGAaTTTC
TACaGGTCTCGCCTCCACCTATCCGGAACCCGAGGATAAGAAGTCGTACATCGAGTCGCACCTGTACAACTACAATTACCAAAGcGCCATGCCGGTTACCTGG
CCGTGGCCGAAGCAGGAGGAACCGGCGAAAACGCGGGAGATTAAGCCCACCCAGCTGCAGGCATAAGCTGCCCCGGAACCAACCGCATTGGGGAgTTTtATTt
CCATCGGCATCAGTTTCCACCAGTATTTATGTTGtCAACGTTATTAGGTCATTTATTTATTTtAACCACCATTGCTCTATTTAGAGATGCTGGGCCCGGAACA
AGTCCTtTTACTtCTACCATCATCCTGCAGGCGTGACCTCGTTGAAAACGGGAATTTGGATACCCACGGCGCCGAAAATAGAGGGCGAGAGATAAACAGAGaG
GgAGAGAGAGAGTGGAAAATCGTAACGATGTTCCTTTTTtATCGTAATAACCCTAAAGTAACCACTCCCTTTACGATTAGCATTAAAAAAAaGGCAGATTAAT
ATGATGATTGGTTGGAACATTATCTCACCACAGTGCTCCCGGACTCGGAAACGAGGTTAAACGAGGTCCGATCGATAGATAAAAAAAAaTCTCTAAAACAAAT
TTCAAAACTAGAAACAAAATTTTGGAATGGCCGTGaCAGCGACTAGCGGAATGTTTGTTTGATTGCGTTCGTCTGGGCTTTGGGCTAAAGTTTAACCGACCGC
GCTGCCACACACTCGATTGTtGTCAAAGTAAAaTAAAACTATTACAAACAAAAAGATGATTAAAAAAaTACTTACCGAGCTGAAGCgaGTCGGTATTCGAGAT
ACTTGCAATAAAATGAACCGTTTTATCAGACACGATAAATGCTCTGGAAGGAGAATGTGAATCGTTGAGTTTTATTtGCTTTCGCAAAACCATTACACCCTAA
GGATGTATCACGCGCGCTCTNNNNNNNNNNNNNNNNNNNNNNNNNNNNNNNNNNNNNNNNNNNNNNNNNNNNNNNNNNNNNNNNNNNNNNNNNNNNNNNN
NNNNNNNNNNNNNNNNNNNNNNNNNNNNNNNNNNNNNNNNNNNNNNNNNCTCTCTCTGCAGTGCCACTATCACAGTAGTTTAGATCTTTGGTGCACGGTT
CACGAGAAACCAGAGCAATCCGAACAGAAAGGA

B. Protein sequence for malic enzyme derived from gene sequence from *Anopheles stephensi*

MVQGIDHLRDPRLNKGLAFTLEERQILGIHGLQPARFKSQEEQLELCRISISRYQEDLNKYLYLVDLQDRNERLFFRLISEDVEKMMPIVYTPTVGLACQKFG
LIYRRPRGLFVTINDRGHVFDVLRNWPEPDVRAIVVTDGE**RILGLGDLGACGMGIPVGKL**ALYTALAGIPPHQCLPIVIDVGTNNKDLLEDPLYIGLRHKRVQ
GKEYDDFIDEFMQAVVK**RYGQNTLIQFEDFGNHNAFRF**LDKY**RNTYCTFNDDIQGTASVAVAGLLASKR**ITNKRISENTFLFLGAGEAAVGIADLVVKAMQAE
GVGLQEA**RDKIWMFDIDGLLAKG**RPEGRLGGHKAYYAKDHAVMKNFADVVKEVKPSVLIGASAAGGAFTPEILQAMGQFNERPIIFALSNPTSKAECTAQAAY
DNTEVRVG**RCIFASGSPFAPVKYGGKT**YITGQGNNAYIFPGVALGVIVTGTHHIPEDMFLIAAQVVADHVCDADLEKGSLYPPLSAI**KECSMDIAVGVTNYAY**
QKGGLASTYPEPEDK**KSYIESHLYNYNYQSAMPVTWPWPKQ**EEPAKTREIKPTQLQA

(in bold, peptides identified by mass spectrometry).

C. Protein sequences producing significant alignments:

```
                                                                   Score (bits) E Value

tr|Q7QB64|Q7QB64_ANOGA Malic enzyme OS=Anopheles gambiae GN=AgaP   831           0.0

>tr|Q7QB64|Q7QB64_ANOGA Malic enzyme OS=Anopheles gambiae  GN=AgaP_AGAP004159 PE=3 SV=5
          Length = 571

 Score = 831 bits (2146), Expect(3) = 0.0
 Identities = 411/419 (98%), Positives = 417/419 (99%)  Frame = +2

Query: 1001 MVQGIDHLRDPRLNKGLAFTLEERQILGIHGLQPARFKSQEEQLELCRISISRYQEDLNK 1180
            MVQGIDHLRDPRLNKGLAFTLEERQILGIHGLQPARFKSQEEQLELCRISISRYQEDLNK
Sbjct: 1    MVQGIDHLRDPRLNKGLAFTLEERQILGIHGLQPARFKSQEEQLELCRISISRYQEDLNK 60

Query: 1181 YLYLVDLQDRNERLFFRLISEDVEKMMPIVYTPTVGLACQKFGLIYRRPRGLFVTINDRG 1360
            YLYLVDLQDRNERLFFRLISEDVEKMMPIVYTPTVGLACQKFGLIYRRPRGLFVTINDRG
Sbjct: 61   YLYLVDLQDRNERLFFRLISEDVEKMMPIVYTPTVGLACQKFGLIYRRPRGLFVTINDRG 120

Query: 1361 HVFDVLRNWPEPDVRAIVVTDGERILGLGDLGACGMGIPVGKLALYTALAGIPPHQCLPI 1540
            HVFDVLRNWPEPDVRAIVVTDGERILGLGDLGACGMGIPVGKLALYTALAGIPPHQCLPI
```

```
Sbjct: 121  HVFDVLRNWPEPDVRAIVVTDGERILGLGDLGACGMGIPVGKLALYTALAGIPPHQCLPI 180

Query: 1541 VIDVGTNNKDLLEDPLYIGLRHKRVQGKEYDDFIDEFMQAVVKRYGQNTLIQFEDFGNHN 1720
            VIDVGTNNKDLLEDPLYIGLRH+RVQGKEYD+FIDEFMQAVVKRYGQNTLIQFEDFGNHN
Sbjct: 181  VIDVGTNNKDLLEDPLYIGLRHQRVQGKEYDEFIDEFMQAVVKRYGQNTLIQFEDFGNHN 240

Query: 1721 AFRFLDKYRNTYCTFNDDIQGTASVAVAGLLASKRITNKRISENTFLFLGAGEAAVGIAD 1900
            AFRFLDKYRNTYCTFNDDIQGTASVAVAG+LA+KRITNKRIS+NTFLFLGAGEAAVGIAD
Sbjct: 241  AFRFLDKYRNTYCTFNDDIQGTASVAVAGMLAAKRITNKRISDNTFLFLGAGEAAVGIAD 300

Query: 1901 LVVKAMQAEGVGLQEARDKIWMFDIDGLLAKGRPEGRLGGHKAYYAKDHAVMKNFADVVK 2080
            LVVKAMQAEG GLQEARDKIWMFDIDGLLAKGRPEGRLGGHKAYYAKDH VMKNFADVVK
Sbjct: 301  LVVKAMQAEGTGLQEARDKIWMFDIDGLLAKGRPEGRLGGHKAYYAKDHGVMKNFADVVK 360

Query: 2081 EVKPSVLIGASAAGGAFTPEILQAMGQFNERPIIFALSNPTSKAECTAQAAYDNTEVRV 2257
            EVKPSVLIGASAAGGAFTPEILQAMGQFNERPIIFALSNPTSKAECTAQAAYDNTEVR+
Sbjct: 361  EVKPSVLIGASAAGGAFTPEILQAMGQFNERPIIFALSNPTSKAECTAQAAYDNTEVRL 419


 Score = 196 bits (499), Expect(3) = 0.0
 Identities = 94/99 (94%), Positives = 97/99 (97%)  Frame = +3

Query: 2316 GRCIFASGSPFAPVKYGGKTYITGQGNNAYIFPGVALGVIVTGTHHIPEDMFLIAAQVVA 2495
            GRCIFASGSPF PV+YGGKT+ITGQGNNAYIFPGVALGVIVTGTHHIPEDMFLIAAQVVA
Sbjct: 420  GRCIFASGSPFPPVQYGGKTFITGQGNNAYIFPGVALGVIVTGTHHIPEDMFLIAAQVVA 479

Query: 2496 DHVCDADLEKGSLYPPLSAIKECSMDIAVGVTNYAYQKG 2612
            DHV +ADLEKGSLYPPLSAIKECSMDIAVGVTNYAYQKG
Sbjct: 480  DHVSEADLEKGSLYPPLSAIKECSMDIAVGVTNYAYQKG 518


 Score = 110 bits (274), Expect(3) = 0.0
 Identities = 49/54 (90%), Positives = 51/54 (94%)  Frame = +1

Query: 2683 GLASTYPEPEDKKSYIESHLYNYNYQSAMPVTWPWPKQEEPAKTREIKPTQLQA 2844
            GLASTYPEPEDKKSYIESHLYNYNYQSAMPVTWPWPKQ E +KTREI PT+LQA
Sbjct: 518  GLASTYPEPEDKKSYIESHLYNYNYQSAMPVTWPWPKQHESSKTREINPTKLQA 571
```
