# Supplementary material for: Mitochondrial NAD+-dependent malic enzyme from Anopheles stephensi: a possible novel target for malaria mosquito control
Source: Malar J. 2011 Oct 26;10:318. doi: 10.1186/1475-2875-10-318 (PMC3228860; doi:10.1186/1475-2875-10-318)
Supplement: Additional file 2 — Modeling results obtained with A. stephensi ME. This file contains the results obtained with the simulation suite named I-TASSER performed with the primary amino acid sequence of A. stephensi ME. [file 1475-2875-10-318-S2.PDF]

Additional file 2

Submitted Sequence

```
>protein
MVQGIDHLRDPRLNKGLAFTLEERQILGIHGLQPARFKSQEEQLELCRISISRYQEDLNK
YLYLVDLQDRNERLFFRLISEDVEKMMPIVYTPTVGLACQKFGLIYRRPRGLFVTINDRG
HVFDVLRNWPEPDVRAIVVTDGERILGLGDLGACGMGIPVGKLALYTALAGIPPHQCLPI
VIDVGTNNKDLLEDPLYIGLRHKRVQGKEYDDFIDEFMQAVVKRYGQNTLIQFEDFGNHN
AFRFLDKYRNTYCTFNDDIQGTASVAVAGLLASKRITNKRISENTFLFLGAGEAAVGIAD
LVVKAMQAEGVGLQEARDKIWMFDIDGLLAKGRPEGRLGGHKAYYAKDHAVMKNFADVVK
EVKPSVLIGASAAGGAFTPEILQAMGQFNERPIIFALSNPTSKAECTAQAAYDNTEVRVG
RCIFASGSPFAPVKYGGKTYITGQGNNAYIFPGVALGVIVTGTHHIPEDMFLIAAQVVAD
HVCDADLEKGSLYPPLSAIKECSMDIAVGVTNYAYQKGGLASTYPEPEDKKSYIESHLYN
YNYQSAMPVTWPWPKQEEPAKTREIKPTQLQA
```

Predicted Secondary Structure

```
                20   40   60   80   100   120   140   160   180   200   220   240   260   280   300   320   340   360   380   400   420   440   460   480   500   520   540   560
Sequence    MVQGIDHLRDPRLNKGLAFTLEERQILGIHGLQPARFKSQEEQLELCRISISRYQEDLNKYLYLVDLQDRNERLFFRLISEDVEKMMPIVYTPTVGLACQKFGLIYRRP[illegible]
Prediction  CCCHHHHHCCCCCCCCCCCHHHHHHCCCCCCCCCCCCHHHHHHHHHHHHHHCCCCHHHHHHHHHHHHHHHHHHHHHHHHHHHHHCCCCCCCHHHHHHHHHHHCCCC[illegible]
Conf.Score  977688862976566468999999975985688986178999999999999984658669899999999864268879998765697466136984799999999874576[illegible]
            Secondary structure elements are shown as H for Alpha helix,S for Beta sheet & C for Coil
```

Predicted Solvent Accessibility

```
                20   40   60   80   100   120   140   160   180   200   220   240   260   280   300   320   340   360   380   400   420   440   460   480   500   520   540   560
Sequence    MVQGIDHLRDPRLNKGLAFTLEERQILGIHGLQPARFKSQEEQLELCRISISRYQEDLNKYLYLVDLQDRNERLFFRLISEDVEKMMPIVYTPTVGLACQKFGLIYRRP[illegible]
Prediction  73312200210210101000361064030200001203313300320151046263203013002302321220001001420430000000000002002301310220[illegible]
            Values range from 0 (buried residue) to 9 (highly exposed residue)
```

Top 5 Models predicted by I-TASSER

Download Model 1	Download Model 2	Download Model 3	Download Model 4	Download Model 5
C-score= 1.985	C-score=-2.659	C-score=-3.301	C-score=-3.236	C-score=-2.941

Estimated accuracy of Model1: 0.99±0.04 (TM-score) 3.5±2.4Å (RMSD) (Read more about C-score of generated models)

Top 10 templates used by I-TASSER

Sec.Str CCCHHHHHCCCCCCCCCCCHHHHHHCCCCCCCCCCCCHHHHHHHHHHHHHHCCCCHHHHHHHHHHHHHHHHHHHHHHHHHHHHHCCCCCC[illegible]

Seq MVQGIDHLRDPRLNKGLAFTLEERQILGIHGLQPARFKSQEEQLELCRISISRYQEDLNKYLYLVDLQDRNERLFFRLISEDVEKMMPIVYT[illegible]

Rank	PDB Hit	Iden1	Iden2	Cov.	Norm. Z-score	Download Align.	Alignment
1	2aw5B	0.59	0.55	0.94	3.87	Download	---GYLLTRNPHLNKDLAFTLEERQQLNIHGLLPPSFNSQEIQVLRVVKNFEHLNSDFDRYLLLMDLQDRNEKLFYRVLTSDIEKFMPIVYT[illegible]
2	1pj3A	0.56	0.52	0.93	4.41	Download	KEKGKPL-LNPRTNKG-AFTLQERQ-LGLQGLLPPKIETQDIQALRFHRNLKK-TSPLEKYIYI-GIQERNEKLFYRILQDDIESL-PIVYT[illegible]
3	1gz4A	0.56	0.52	0.93	10.01	Download	-EKGKP-LLNPRTNKG-AFTLQERQ-LGLQGLLPPKIETQDIQALRFHRNLK-KTSPLEKYIY-IGIQERNEKLFYRILQDDI-ESLPIVYT[illegible]
4	1llqA	0.53	0.53	0.98	5.45	Download	KKRSAELLKEPRLNKGMGFSLYERQYLGLHGLLPPAFMTQEQQAYRVITKLREQPNDLARYIQLDGLQDRNEKLFYRVVCDHVKELMPIVYT[illegible]
5	2aw5B	0.59	0.55	0.94	7.59	Download	---GYLLTRNPHLNKDLAFTLEERQQLNIHGLLPPSFNSQEIQVLRVVKNFEHLNSDFDRYLLLMDLQDRNEKLFYRVLTSDIEKFMPIVYT[illegible]
6	1pj3A	0.56	0.52	0.93	3.88	Download	KEKGKPL-LNPRTNKG-AFTLQERQ-LGLQGLLPPKIETQDIQALRFHRNLKK-TSPLEKYIYI-GIQERNEKLFYRILQDDIESL-PIVYT[illegible]
7	1o0sA	0.54	0.53	0.98	24.14	Download	KKRSAELLKEPRLNKGMGFSLYERQYLGLHGLLPPAFMTQEQQAYRVITKLREQPNDLARYIQLDGLQDRNEKLFYRVVCDHVKELMPIVYT[illegible]
8	1llqA	0.54	0.53	0.98	11.82	Download	KKRSAELLKEPRLNKGMGFSLYERQYLGLHGLLPPAFMTQEQQAYRVITKLREQPNDLARYIQLDGLQDRNEKLFYRVVCDHVKELMPIVYT[illegible]
9	1gz4A	0.56	0.52	0.93	10.44	Download	-EKGKP-LLNPRTNKG-AFTLQERQ-LGLQGLLPPKIETQDIQALRFHRNLK-KTSPLEKYIY-IGIQERNEKLFYRILQDDI-ESLPIVYT[illegible]
10	2aw5B	0.59	0.55	0.94	6.67	Download	---GYLLTRNPHLNKDLAFTLEERQQLNIHGLLPPSFNSQEIQVLRVVKNFEHLNSDFDRYLLLMDLQDRNEKLFYRVLTSDIEKFMPIVYT[illegible]

(a) All the residues are colored in black; however, those residues in template which are identical to the residue in the query sequence are highlighted in color. Coloring scheme is based on the property of amino acids, where polar are brightly coloured while non-polar residues are colored in dark shade. (more about the colors used)

(b) Rank of templates represents the top ten threading templates used by I-TASSER.

(c) Ident1 is the percentage sequence identity of the templates in the threading aligned region with the query sequence.

(d) Ident2 is the percentage sequence identity of the whole template chains with query sequence.

(e) Cov. represents the coverage of the threading alignment and is equal to the number of aligned residues divided by the length of query protein.

(f) Norm. Z-score is the normalized Z-score of the threading alignments. Alignment with a Normalized Z-score >1 mean a good alignment and vice versa.

(g) Download Align. provides the 3D structure of the aligned regions of the threading templates.

(h) The top 10 alignments reported above (in order of their ranking) are from the following threading programs:

1: MUSTER 2: HHSEARCH 3: SP3 4: PROSPECT2 5: PPA-I 6: HHSEARCH I 7: COMA 8: FUGUE 9: SPARKS 10: SAM

10 proteins in PDB which are structurally closest to the first I-TASSER model (identified by TM-align)

Rank	TM-score	RMSD[a]	IDEN[a]	Cov.	PDB Hit	Structural alignment using TM-align
1	0.9645	1.43	0.53	0.99	1o0sA<br>Model1	SVAHHEDVYSHNLPPMDEKEMALYKLYRPERVTPKKRSAELLKEPRLNKGMGFSLYERQYLGLHGLLPPAFMTQEQQAYRVITKLREQPNDLARYIQLDGLQ[illegible]<br>--------------------------------MVQGIDHLRDPRLNKGLAFTLEERQILGIHGLQPARFKSQEEQLELCRISISRYQEDLNKYLYLVDLQ[illegible]
2	0.9244	0.93	0.59	0.94	2aw5B<br>Model1	---GYLLTRNPHLNKDLAFTLEERQQLNIHGLLPPSFNSQEIQVLRVVKNFEHLNSDFDRYLLLMDLQDRNEKLFYRVLTSDIEKFMPIVYTPTVGLACQQY[illegible]<br>MVQGIDHLRDPRLNKGLAFTLEERQILGIHGLQPARFKSQEEQLELCRISISRYQEDLNKYLYLVDLQDRNERLFFRLISEDVEKMMPIVYTPTVGLACQKF[illegible]
3	0.9211	1.06	0.56	0.94	1ef1A<br>Model1	IKEKGKPL-LNPRTNKG-AFT[illegible]<br>-MVQGIDHLRDPRLNKGLAFT[illegible]
4	0.6094	3.33	0.22	0.67	3nv9A<br>Model1	MAQL[illegible]<br>----MVQGIDHLRDPRLNKGL[illegible]
5	0.6020	2.73	0.23	0.65	2dvmA<br>Model1	[illegible]<br>MVQGIDHLRDPRLNKGLAFTL[illegible]
6	0.5466	2.36	0.23	0.58	2haeA<br>Model1	[illegible]<br>MVQGIDHLRDPRLNKGLAFTL[illegible]
7	0.5357	3.01	0.21	0.59	2a9fA<br>Model1	L[illegible]<br>-MVQGIDHLRDPRLNKGLAFT[illegible]
8	0.4629	6.49	0.09	0.66	2bmaA<br>Model1	L-----------------KDK[illegible]<br>-MVQGIDHLRDPRLNKGLA--[illegible]
9	0.4499	5.97	0.10	0.63	1k89A<br>Model1	S[illegible]<br>-MVQGIDHLRDPRLNKGLAFT[illegible]
10	0.4425	6.04	0.09	0.62	3aoeA<br>Model1	[illegible]<br>MVQGIDHLRDPRLNKGLAFTL[illegible]

(a) Residue pairs that are structurally aligned by TM-align program are highlighted in color. Coloring scheme is based on the property of amino acids, where polar are brightly coloured while non-polar residues are colored in dark shade.(more about the colors used)

(b) Ranking of proteins is based on TM-score of the structural alignment between Model1 and the PDB structures in our template library.

(c) RMSD[a] is the RMSD between residues that are structurally aligned by TM-align.

(d) IDEN[a] is the percentage sequence identity in the structurally aligned region.

(e) Cov. represents the coverage of the alignment by TM-align and is equal to the number of structurally aligned residues divided by length of the model.

Function Prediction

Predicted EC Numbers

Rank	TM-score	RMSD[a]	IDEN[a]	Cov.	EC-Score	PDB Hit	EC No.
1	0.9508	1.17	0.60	0.97	3.1352	1gq2A	1.1.1.40
2	0.9244	0.93	0.59	0.94	3.0532	2aw5B	1.1.1.40
3	0.9440	1.10	0.56	0.96	2.9908	1gz3A	1.1.1.38
4	0.9645	1.43	0.53	0.99	2.9372	1o0sA	1.1.1.38
5	0.6020	2.73	0.23	0.65	1.2213	2dvmA	1.1.1.38

(a) Ranking is based on EC-score, which is a confidence score for the Enzyme Classification (EC) Number prediction.

(b) RMSD[a] is the RMSD between models and the PDB structure in the structurally aligned regions by TM-align.

(c) IDEN[a] is percentage sequence identity in the structurally aligned region.

(d) Cov. represents the coverage of the alignment and is equal to the number of structurally aligned residues divided by length of model.

(e) EC-Score is defined based on the C-score of the structure prediction and similarity of the model with known enzyme structures, as identified using both global and local structural alignment programs. The global similarity score uses TM-score, IDEN[a],RMSD[a] and Cov. of the structural alignment by TM-align, while the local match compares the structural and chemical similarity of local spatial motifs in the model with known catalytic site of enzymes. A prediction with a EC-score >1.1 signifies a prediction with high confidence (upto 3 digit numbers of EC) and vice versa (For details, see Inferring protein function by global and local similarity of structural analogs, 2010, submitted).

Predicted GO terms

Rank	TMscore	RMSD[a]	IDEN[a]	Cov.	PDB Hit	Fh-Score	Associated GO Terms
1	0.9270	1.16	0.61	0.94	1gq2P	3.07	GO:0004470 GO:0004473 GO:0046872 GO:0051287 GO:0006108 GO:0009987 GO:0055114 GO:0005622 GO:0005623 GO:0005737
2	0.9187	0.94	0.59	0.93	2aw5C	3.04	GO:0004470 GO:0004473 GO:0009055 GO:0030145 GO:0030554 GO:0043169 GO:0043531 GO:0050661 GO:0051287 GO:0005975 GO:0006108 GO:0006733 GO:0006739 GO:0006741 GO:0009058 GO:0009108 GO:0009110 GO:0009165 GO:0009725 GO:0009743 GO:0009987 GO:0042364 GO:0044249 GO:0055114 GO:0005622 GO:0005623 GO:0005634 GO:0005730 GO:0005737 GO:0005829 GO:0031974 GO:0043229 GO:0043232 GO:0044428
3	0.9532	1.22	0.54	0.97	1o0sB	2.95	GO:0004470 GO:0016619 GO:0046872 GO:0051287 GO:0006108 GO:0009987 GO:0055114 GO:0005622 GO:0005623 GO:0005737 GO:0005739 GO:0005759 GO:0031974 GO:0043229 GO:0044429 GO:0044444
4	0.9198	1.07	0.56	0.93	1gr6B	2.93	GO:0004470 GO:0004471 GO:0009055 GO:0016619 GO:0046872 GO:0051287 GO:0006108 GO:0009987 GO:0055114 GO:0005622 GO:0005623 GO:0005737 GO:0005739 GO:0005759 GO:0031974 GO:0043229 GO:0044429 GO:0044444
5	0.4644	0.80	0.62	0.47	1gq2P	1.59	GO:0004470 GO:0004473 GO:0046872 GO:0051287 GO:0006108 GO:0009987 GO:0055114 GO:0005622 GO:0005623 GO:0005737
6	0.4384	0.59	0.65	0.44	2aw5C	1.57	GO:0004470 GO:0004473 GO:0009055 GO:0030145 GO:0030554 GO:0043169 GO:0043531

							GO:0050661 GO:0051287 GO:0005975 GO:0006108 GO:0006733 GO:0006739 GO:0006741 GO:0009058 GO:0009108 GO:0009110 GO:0009165 GO:0009725 GO:0009743 GO:0009987 GO:0042364 GO:0044249 GO:0055114 GO:0005622 GO:0005623 GO:0005634 GO:0005730 GO:0005737 GO:0005829 GO:0031974 GO:0043229 GO:0043232 GO:0044428
7	0.4857	0.75	0.54	0.49	2aw5C	1.55	GO:0004470 GO:0004473 GO:0009055 GO:0030145 GO:0030554 GO:0043169 GO:0043531 GO:0050661 GO:0051287 GO:0005975 GO:0006108 GO:0006733 GO:0006739 GO:0006741 GO:0009058 GO:0009108 GO:0009110 GO:0009165 GO:0009725 GO:0009743 GO:0009987 GO:0042364 GO:0044249 GO:0055114 GO:0005622 GO:0005623 GO:0005634 GO:0005730 GO:0005737 GO:0005829 GO:0031974 GO:0043229 GO:0043232 GO:0044428
8	0.4524	0.98	0.59	0.46	1qr6B	1.48	GO:0004470 GO:0004471 GO:0009055 GO:0016619 GO:0046872 GO:0051287 GO:0006108 GO:0009987 GO:0055114 GO:0005622 GO:0005623 GO:0005737 GO:0005739 GO:0005759 GO:0031974 GO:0043229 GO:0044429 GO:0044444
9	0.4759	1.01	0.50	0.48	1o0sB	1.43	GO:0004470 GO:0016619 GO:0046872 GO:0051287 GO:0006108 GO:0009987 GO:0055114 GO:0005622 GO:0005623 GO:0005737 GO:0005739 GO:0005759 GO:0031974 GO:0043229 GO:0044429 GO:0044444
10	0.6015	2.73	0.23	0.65	2dvmD	1.22	GO:0016616 GO:0051287 GO:0008152

Consensus Prediction of Gene Ontology terms

Molecular Function		Biological Process		Cellular Location	
GO term	GO-Score	GO term	GO-Score	GO term	GO-Score
GO:0051287	1.849	GO:0009987	1.669	GO:0005737	2.063
GO:0004470	1.669	GO:0055114	1.669	GO:0005623	2.063
GO:0046872	1.587	GO:0006108	1.669	GO:0005622	2.063
GO:0009055	0.891	GO:0051188	0.473	GO:0043229	1.597
GO:0016619	0.838	GO:0006769	0.473	GO:0031974	1.597
GO:0004473	0.831	GO:0006739	0.473	GO:0005759	0.982
GO:0004471	0.501	GO:0009110	0.473	GO:0044429	0.982
GO:0000166	0.473	GO:0019362	0.473	GO:0005739	0.982
GO:0030145	0.473	GO:0009165	0.473	GO:0043232	0.615
GO:0046914	0.473	GO:0006741	0.473	GO:0005730	0.615

(a) Ranking in the first table is based on a function prediction score (Fh-score), which is calculated based on the C-score of the structure prediction and the TM-score, IDEN[a], RMSD[a] and Cov. of the structural alignment by TM-align between the predicted model and the PDB structures (For details, see Inferring protein function by global and local similarity of structural analogs, 2010, submitted).

(b) RMSD[a] is the RMSD between models and the PDB structure in the structurally aligned regions by TM-align.

(c) IDEN[a] is the percentage sequence identity in the structurally aligned region.

(d) Cov. represents the coverage of the alignment and is equal to the number of structurally aligned residues divided by length of model.

(e) A consensus prediction of GO terms is derived from the structural analogs that have an Fh-score of >=1.0. The GO-Score associated with each prediction is defined as the average weight of the GO term, where the weights are assigned based on the Fh-score of the template from which the GO term is derived. A prediction with a GO-score >0.5 signifies a prediction with high confidence and vice versa.

Predicted Binding Site

Identified stuctural analogs with similar binding site:

Rank	PDB Hit	TM-score	RMSDa	IDENa	Cov.	BS-score	Lig. Name	Download Complex	Predicted binding site residues in the model
1	1gq2G	0.9506	1.1700	0.6030	0.9685	1.6670	MN	Download	162,234,235,258
2	1gq2G	0.9506	1.1700	0.6030	0.9685	1.6410	OXL	Download	91,144,146,162,234,235,258,399,446,447
3	1gz4C	0.9436	1.0500	0.5570	0.9580	1.5580	TTN	Download	91,144,146,162,234,235,258,399,447
4	1pj2B	0.9471	0.9800	0.5570	0.9598	1.5328	MLT	Download	91,92,144,146,162,234,235,258,399,446,447
5	1gq2G	0.9506	1.1700	0.6030	0.9685	1.7925	NAP	Download	144,146,147,238,258,262,289,290,291,292,293,294,323,324,325,370,371,372,373,397,398,399,445,446,447
6	1pj2B	0.9471	0.9800	0.5570	0.9598	1.6550	NAI	Download	133,135,171,172,173,176,459,460,462,523,533,537,538
7	1gz3A	0.9440	1.1000	0.5570	0.9598	1.5885	FUM	Download	43,46,67,70,74
8	1o0sB	0.9532	1.2200	0.5410	0.9720	1.5111	TTN	Download	38,43,46,47,70,74
9	1eflC	0.9448	1.0700	0.5570	0.9598	1.9962	MG	Download	162,234,235,258
10	1gz3A	0.9440	1.1000	0.5570	0.9598	1.5762	OXL	Download	91,138,146,162,234,235,258,399,446,447

Click on the radio buttons to visualize predicted binding sites and residues.

(a) Ranking of the predicted binding sites is based on the size of the clusters formed after local superposition of ligands of the templates onto the query structure.

(b) BS-score is a measure of local sequence & structure similarity between template's binding site and predicted binding site in the query structure. Based on large scale benchmarking analysis, binding site predictions with BS-score>1.1 signify predictions with high confidence.

(c) RMSD[a] the RMSD between residues that are structurally aligned by TM-align.

(d) IDEN[a] is the percentage sequence identity in the structurally aligned region.

(e) Cov. represents the coverage of the alignment by TM-align and is equal to the number of structurally aligned residues divided by length of the model.

(f) For details, see *COFACTOR: protein-ligand binding site predictions by global structure match and local geometry refinement.* (In preparation)

☐Spin On/Off ☐Spin On/Off

Please cite following articles when you use the I-TASSER server:

1. Ambrish Roy, Alper Kucukural, Yang Zhang. I-TASSER: a unified platform for automated protein structure and function prediction. Nature Protocols, vol 5, 725-738 (2010).
2. Yang Zhang. Template-based modeling and free modeling by I-TASSER in CASP7. Proteins, vol 69 (Suppl 8), 108-117 (2007).
