# Supplementary material for: Mitochondrial NAD+-dependent malic enzyme from Anopheles stephensi: a possible novel target for malaria mosquito control
Source: Malar J. 2011 Oct 26;10:318. doi: 10.1186/1475-2875-10-318 (PMC3228860; doi:10.1186/1475-2875-10-318)
Supplement: Additional file 3 — Additional information on the nomenclature of proteins listed under Figure 4. This file contains all additional information (full name, short name, E.C. number, and species) in regards to the nomenclature of ME shown under Figure 4. [file 1475-2875-10-318-S3.PDF]

Additional file 3

Human $NADP^+$ ME-3
MAON_HUMAN
RecName: Full=$NADP^+$-dependent malic enzyme, mitochondrial
Short=NADP-ME
EC=1.1.1.40
AltName: Full=Malic enzyme 3
Flags: Precursor_ [*Homo sapiens* (Human)]

Human NAD^+ ME-2
MAOM_HUMAN
RecName: Full=NAD^+-dependent malic enzyme, mitochondrial
Short=NADME
EC=1.1.1.38
AltName: Full=Malic enzyme 2
Flags: Precursor_ [*Homo sapiens* (Human)]

Human $NADP^+$ ME-1
MAOX_HUMAN
RecName: Full=$NADP^+$-dependent malic enzyme
Short=$NADP^+$-ME
EC=1.1.1.40
AltName: Full=Malic enzyme 1
[*Homo sapiens* (Human)]

Mosquito ME
Protein sequence derived from genomic sequence of *Anopheles stephensi* ME (Additional file 2).

Bacterial NAD^+ ME
MAOX_GEOSE
RecName: Full=NAD^+-dependent malic enzyme
Short=NAD^+-ME
EC=1.1.1.38
[*Geobacillus stearothermophilus (Bacillus stearothermophilus*)]

Nematode NAD ME-2
MAOM_ASCSU
RecName: Full=NAD^+-dependent malic enzyme, mitochondrial
Short=NADME
EC=1.1.1.38
Flags: Precursor_ Fragment_ [*Ascaris suum* (Pig roundworm) (*Ascaris lumbricoides*)]

Plant NADP ME-4
162463046_162463047 *Zea mays* $NADP^+$ malic enzyme4 (me4), mRNA.

Plant chl NADP
MAOC_MAIZE
RecName:
Full=$NADP^+$-dependent malic enzyme, chloroplastic
Short=NADPME
EC=1.1.1.40
Flags: Precursor_ [*Zea mays* (Maize)]

Plant NADP ME-2
162462659_162462660 *Zea mays* $NADP^+$-dependent malic enzyme (me2), mRNA.
